# Artificial Cognition for Detection of Mental Disability: A Vision Transformer Approach for Alzheimer’s Disease

**DOI:** 10.3390/healthcare11202763

**Published:** 2023-10-18

**Authors:** Maram Fahaad Almufareh, Samabia Tehsin, Mamoona Humayun, Sumaira Kausar

**Affiliations:** 1 Department of Information Systems, College of Computer and Information Sciences, Jouf University, Sakakah 72388, Saudi Arabia; mfalmufareh@ju.edu.sa; 2 Department of Computer Science, Bahria University, Islamabad 44000, Pakistan; stehseen.buic@bahria.edu.pk (S.T.); sumairakausar.buic@bahria.edu.pk (S.K.)

**Keywords:** mental disability, diagnosis, Alzheimer’s disease, medical image analysis, vision transformer

## Abstract

Alzheimer’s disease is a common neurological disorder and mental disability that causes memory loss and cognitive decline, presenting a major challenge to public health due to its impact on millions of individuals worldwide. It is crucial to diagnose and treat Alzheimer’s in a timely manner to improve the quality of life of both patients and caregivers. In the recent past, machine learning techniques have showed potential in detecting Alzheimer’s disease by examining neuroimaging data, especially Magnetic Resonance Imaging (MRI). This research proposes an attention-based mechanism that employs the vision transformer approach to detect Alzheimer’s using MRI images. The presented technique applies preprocessing to the MRI images and forwards them to a vision transformer network for classification. This network is trained on the publicly available Kaggle dataset, and it illustrated impressive results with an accuracy of 99.06%, precision of 99.06%, recall of 99.14%, and F1-score of 99.1%. Furthermore, a comparative study is also conducted to evaluate the performance of the proposed method against various state-of-the-art techniques on diverse datasets. The proposed method demonstrated superior performance, outperforming other published methods when applied to the Kaggle dataset.

## 1. Introduction

Alzheimer’s disease (AD) is a condition of neurodegeneration that is distinguished by the gradual deterioration in memory, cognitive skills, and reasoning capabilities. It is a leading cause of dementia in the elderly, with symptoms typically starting in mid- to late life. The underlying mechanisms of AD are not yet fully understood; however, it is hypothesized that AD arises from a blend of genetic and environmental elements. The brain of an Alzheimer’s patient experiences the loss of neurons and connections between neurons, leading to a decline in cognitive function. Presently, no definitive remedy for AD has been identified, and there are multiple treatment options available that can aid in symptom management and decelerate the progression of the condition.

The timely detection of AD holds significant importance because it can help individuals and their families prepare for the future, access treatments and support services, and plan for long-term care. However, early detection can pose challenges as the symptoms of AD can resemble those of other conditions and may even be considered a normal part of the aging process. In numerous countries, including the United States, AD is officially recognized as a disability. As per the Americans with Disabilities Act (ADA), a disability refers to a substantial impairment in one or more crucial life activities arising from a mental or physical disability, such as mobility, vision, hearing, or cognitive function. Given that AD can substantially impede a person’s capacity to think, remember, and communicate effectively—key aspects of daily life—it is regarded as a disability. This limitation can make it challenging for individuals with AD to perform tasks they were previously capable of, such as managing finances or driving. Moreover, AD has the potential to affect an individual’s ability to involve themselves in social activities, leading to social isolation and a decline in overall quality of life. Under the ADA, people with AD are entitled to reasonable accommodations in the workplace, such as modified work schedules or job duties, as well as access to public accommodations and services, such as transportation or housing. Moreover, individuals diagnosed with AD might be eligible for Social Security Disability Insurance (SSDI) or Supplemental Security Income (SSI) benefits if their condition hinders their ability to engage in gainful employment and sustain a livelihood.

The diagnostic process for AD entails a thorough assessment that encompasses a medical history and physical examination, a neurological evaluation, cognitive and psychological assessments, brain imaging studies such as Magnetic Resonance Imaging (MRI) or positron emission tomography (PET) scans, and laboratory tests to exclude other disorders with similar symptoms. Structural Magnetic Resonance Imaging (sMRI) is extensively utilized to study progressive neurological impairments. This non-invasive technique employs radio waves and strong magnetic fields to analyze brain anatomical changes, ensuring a high level of spatial resolution and providing a painless imaging method [1]. The field of Artificial Intelligence (AI) is progressing at a swift pace, exhibiting the capacity to transform numerous sectors. AI could transform many industries. The progress that has been made in AI is very encouraging from a scientific perspective. AI is having a significant impact on healthcare, particularly in the field of medical diagnosis. AI is being used to create systems that can analyze large amounts of medical data and identify patterns that may be indicative of disease. This research emphasizes the use of AI techniques to diagnose AD using brain MRIs. The novelty of the presented technique lies in its application of the vision transformer approach, originally designed for natural language processing tasks, to detect Alzheimer’s disease using MRI images. By employing an attention-based mechanism within the vision transformer network, the method showcases remarkable efficacy in accurately diagnosing Alzheimer’s. Furthermore, the paper includes a comprehensive comparative study that demonstrates the superiority of the proposed approach over various state-of-the-art techniques when applied to the Kaggle dataset. This novel combination of the vision transformer and attention-based mechanism for Alzheimer’s detection contributes to the growing body of research in the field of neuroimaging and machine learning.

The first section of this article represents the introduction of the domain and research problem. After the introduction, this paper provides an analysis of related state-of-the-art work and identifies gaps in the research. The third section describes the methodology, including the proposed algorithms and techniques for data acquisition, preprocessing, feature extraction, and classification. The results and analysis section presents the research findings, including evaluation metrics used to assess the model’s performance and analysis of the results. Furthermore, this section compares the achieved results with other prominent studies. This paper is concluded in the last section by summarizing the main contributions and the future research directions.

## 2. Literature Review

The advancements achieved in AI are highly promising from a scientific standpoint [2,3,4]. The area of computer-aided diagnosis is advancing rapidly [5,6,7]. A variety of studies have examined the use of AI, machine learning, and image analysis techniques to examine brain scans and identify AD. Specifically, AI systems can be trained to identify patterns in MRI, fMRI, and other types of scans that are characteristic of AD such as the shrinkage or dilation in specific areas of the brain.

### 2.1. Machine Learning Methods

There are many methods in the literature that use traditional machine learning models for the detection of Alzheimer’s. Kloppel et al. [8] proposed a technique to lessen the dimensionality of input features. Furthermore, the detection of AD patients can be accomplished through the utilization of a support vector machine (SVM) algorithm. A poly-modal discriminator for PET and MRI images employing a random forest predictor was proposed in Gray et al.’s study [9]. In another work by Neati et al. [10], a union of SVM and kernel principal component analysis (PCA) was introduced for dimensionality reduction and extraction of information from MRI images, achieving 92.5% accuracy on the OASIS datasets.

In their study [10], researchers aimed to achieve 100% accuracy by introducing wavelet entropy and the biography optimization engine. They employed a six-fold cross-validation technique on sixty-four brain images. Another approach for improving feature extraction and selection accuracy on datasets of AD and normal control patients was developed by [11]. They utilized a matrix of gray-level occurrences and an approach based on voxel-based morphometry. El-Dahshan et al. [12] presented two classifiers for feature representation and selection of weighted MRI images obtained from the Harvard Medical School and facilitated the multi-classification of AD.

Wavelet entropy and the multilayer perceptron were employed by Wang et al. [13] to develop a new AD detection system. The technique was tested on three-dimensional volumetric data by selecting optimum slices. Li et al. [14] obtained ninety-three 3D features from MRI and PET images using ROI, and subsequently conducted PCA.

### 2.2. Deep Learning Methods

Deep learning-based techniques have shown promising results in detecting AD [15]. These methods typically involve using neural network algorithms to analyze medical imaging data like MRI or PET scans, and identify characteristic patterns associated with AD.

One example of a deep learning method used for AD detection is a Convolutional Neural Network (CNN), which has been used to analyze structural MRI scans and predict the presence of AD. In the realm of deep learning, scholars can devise their own framework by relying on Convolutional Neural Networks (CNNs), which have demonstrated remarkable success. Numerous refined CNN models have surfaced and offer a proficient means of detecting AD, like VGGNet, AlexNet, ResNet, DenseNet, and Inception [16].

In another study [17], a high-performance multiple sclerosis classification model was developed. AlexNet served as the foundational model, and transferred learning was applied to adapt it for the specific task of classifying multiple-sclerosis brain images. Various configurations of transfer learning were tested, involving the transfer and replacement of different numbers of layers. Another study is presented to automate the detection of pathological brain regions in Magnetic Resonance Imaging (MRIs) images using a deep learning structure combined with transfer learning [18]. Initially, a pre-trained AlexNet structure was obtained, and then the parameters of the last three layers were replaced with random weights, while the remaining parameters retained their pre-trained values.

Another example is the utilization of Recurrent Neural Networks (RNNs) to analyze functional MRI data and predict cognitive decline in AD patients.

In this study [19], the researchers aim to predict the clinical diagnosis, cognition, and ventricular volume of individuals based on multimodal AD markers and clinical diagnosis data from one or more time points. To achieve this, a minimal Recurrent Neural Network (minimalRNN) model was proposed and applied to longitudinal data from The Alzheimer’s Disease Prediction Of Longitudinal Evolution (TADPOLE) challenge.

The researchers devise an automated classification method capable of effectively handling EEG data [20]. The study demonstrates the use of the RNN robust principal component analysis (RPCA) algorithm for AD detection. In another study [21], neuropsychological measures and MRI biomarkers are derived and subsequently fed into an RNN. The RNN utilizes the long short-term memory (LSTM) architecture, and the proposed model aims to forecast the biomarkers (feature vectors) of patients at 6, 12, 18, 21, 24, and 36 months into the future.

Ren et al. [22] proposed three classifiers—SBPCNNs with a single slice, SACNNs with simple assembly, and MSCNNs with multiple slices—to observe the model using a reduced number of slices, achieving over 90% performance in AD detection. Zhang et. al. [23] presented a deep neural network framework for sMRI gray matter slicing, using the attention approach to enhance characteristic information and improve accuracy by 1–8% in comparison to contemporary techniques.

Suk et al. [24] used resting-state functional Magnetic Resonance Imaging (rs-fMRI) to select Regions of Interest (ROIs), and then trained a deep model for every ROI. They also proposed an auto-encoder to uncover hierarchical connections between different ROIs.

In [25], Shi et al. proposed a stacked denoising sparse auto-encoder that utilized an SVM for classification. Subsequently, ref. [26] employed a deep belief network composed of a layered Restricted Boltzmann Machine for AD detection, and the results demonstrated superior performance compared to the SVM. In their work, Shakeri et al. [27] employed a deep probabilistic autoencoder to acquire low-dimensional feature portrayal from hippocampal morphological variations. Another study [28] introduced a neural network architecture with multi-scale depth for the purpose of AD diagnosis. Their approach utilized the basic-level patch features extracted from PET images.

The study in [29] utilizes the vision transformer architecture to automatically detect Alzheimer’s patients from healthy controls. The vision transformer architecture is chosen for its ability to effectively capture global or long-range relationships among image features. To improve the network’s performance, frequency and image domain features are integrated since MRI data are acquired in the frequency domain before being transformed into images. Another study [30] introduces a novel approach to AD classification using a Dual-Input Convolution Encoder Network (DICE-net). The proposed method involves denoising the EEG data, followed by the extraction of band power and coherence features. These extracted features are then fed into the DICE-net, which comprises convolution, transformer encoder, and feedforward layers.

A very deep convolutional network is designed by Islam et al. [31], and the performance is demonstrated on the Open Access Series of Imaging Studies (OASIS) database.

Deep learning techniques have shown great promise in detecting AD. In most of the cases, they have outperformed other image analysis methods and even human experts in certain instances. However, there are a few limitations to deep learning methods for AD detection. Notwithstanding these limitations, deep learning methods have the capacity to revolutionize the diagnosis and treatment of AD.

## 3. Proposed Methodology

### 3.1. Data

**Figure 1:**
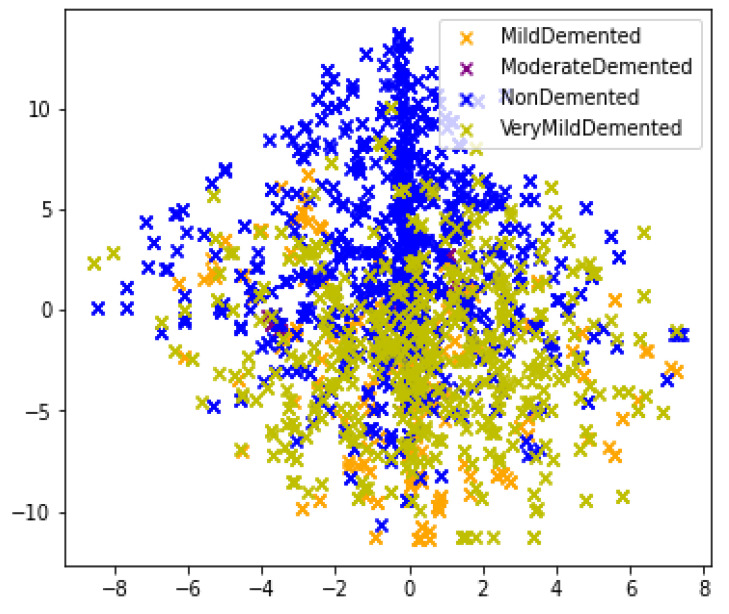
T-SNE visualization of the dataset.

The dataset for AD was obtained from the Open Access Series of Imaging Studies-1 (OASIS-1) [32,33]. The OASIS is a project with the goal of providing the scientific community with open access to neuroimaging datasets of the brain. Its objective is to promote advancements in both basic and clinical neuroscience by gathering and openly sharing neuroimaging datasets, thus fostering potential breakthroughs in the field. It consists of cross-sectional MRI data from young, middle-aged, non-demented, and demented older adults. It comprises 80k MRI images categorized into quaternary classes: Mild-Demented (MID), Moderate Demented (MOD), Non-Demented (ND), and Very Mild Demented (VMD). Patient classification was performed based on the provided metadata and Clinical Dementia Rating (CDR) values. CDR is based on Mini-Mental State Exam (MMSE) for Alzheimer’s [34]. The dataset undergoes partitioning in an 80–20 ratio, with 80% of the data allocated for training and the remaining 20% designated for validation purposes. The 80:20 split validation offered a reasonable baseline to showcase the performance gain achieved by the advanced architecture compared to previous models. This setup allowed us to directly compare against prior studies, providing a benchmark for the community. Many medical researchers have used this split validation approach [35,36,37,38]. The images in the dataset have a size of 176 × 208 pixels. Figure 1 shows the distribution of data in feature space for various classes.

### 3.2. Data Preparation

Data augmentation is a technique used to increase the size of a training dataset by creating new data points from existing data points. This can help to improve the performance of deep learning models by reducing overfitting. This research uses rotation and flipping techniques to enhance the size of training data [39]. Rotation involves rotating an image by a certain angle γ to create a new image. The mathematical representation of rotation can be given as follows:

(1) $$Img_{rotated}\left(s,t\right)=Img\left(s.cos\left(\gamma \right)-t.sin\left(\gamma \right),s.sin\left(\gamma \right)+t.cos\left(\gamma \right)\right)$$

where Img(s,t) represents the original image, $Img_{rotated}\left(s,t\right)$ represents the rotated image, and γ represents the angle of rotation. Flipping involves horizontally or vertically mirroring an image to create a new image. The mathematical representation of flipping can be given as follows:

(2) $$Img_{Vflipped}\left(s,t\right)=Img\left(s,H-t\right)$$

(3) $$Img_{Hflipped}\left(s,t\right)=Img\left(W-s,t\right)$$

where Img(s,t) represents the original image, $Img_{Vflipped}\left(s,t\right)$ represents the vertically flipped image, and $Img_{Hflipped}\left(s,t\right)$ represents the horizontally flipped image. The variables *W* and *H* mean the no. of columns and rows of the image, correspondingly.

### 3.3. Preprocessing

The input image is normalized first. Image normalization is a process of rescaling pixel values of an image so that they lie within a certain range of values [40]. The purpose of normalization is to standardize the intensity values of an image to facilitate comparison and analysis across different images. Mathematically, image normalization can be represented:

(4) $$Img_{norm}=\left(Img-min\left(Img\right)\right)/\left(max\left(Img\right)-min\left(Img\right)\right)$$

where Img is the original image, $Img_{norm}$ is the normalized image, and min(Img) and max(Img) represent the lowest and highest pixel values of Img, respectively. The resulting $Img_{norm}$ values will be in the range of 0 to 1. After normalization, the image is treated for bias field correction. Bias field correction is a technique used to correct for intensity inhomogeneities or non-uniformities in the MRI signal. These non-uniformities can be caused by various factors such as magnetic field inhomogeneities, patient motion, and hardware issues. Mathematically, the bias field correction can be represented as follows:

(5) $$I_{corr}=Img_{norm}/B$$

where $Img_{norm}$ is the original image, *B* is the estimated bias field calculated by the Non-Parametric Non-Uniformity Normalization (N3) algorithm [41], and $I_{corr}$ is the corrected image.

### 3.4. Feature Extraction

The proposed methodology uses transformer networks for feature extraction. Moreover, it uses the concept of transfer learning to deal with the data scarcity issue.

**Figure 2:**
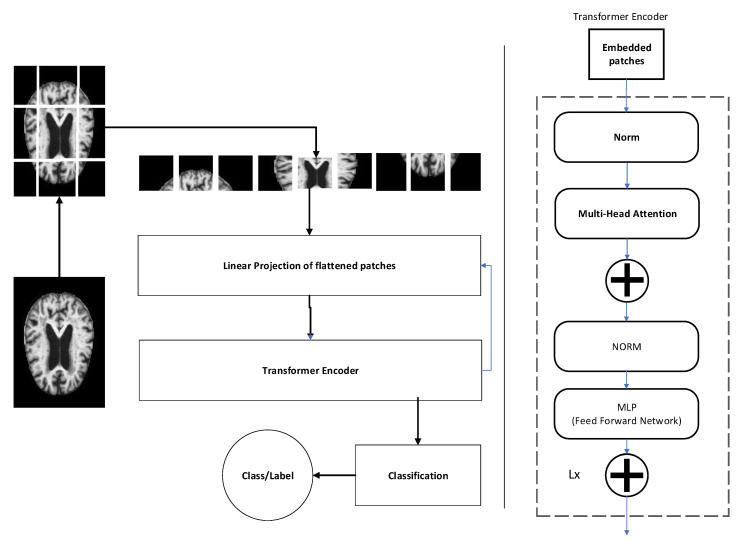
Diagrammatic illustration of proposed feature extraction and classification method.

Given an input image Img$\;\in R^{HxWxC}$ with label as the ground truth value of v binary labels $lab_{1},lab_{2},\ldots \ldots lab_{v}$, where $lab_{1}\in 0,1$, v=4 represents four different classes of Alzheimer patients. The aim of this methodology is to structure a classification function, cf, to predict a set of labels provided for an image so that $l\hat{a}b=cf\left(Img\right)$. A vision transformer refers to a specific neural network architecture that draws inspiration from the transformer architecture, originally created for tasks in natural language processing, stands as its initial application [42]. In a vision transformer, the input is typically an image, and the goal is to perform tasks like object identification, segmentation, or classification [43,44]. Figure 2 shows architectural details of the proposed methodology. The first step in the vision transformer is to convert the input image into a sequence of tokens suitable for the transformer architecture. Subsequent paragraphs explain each module of the architecture in detail. The first step is the tokenization of the input image. Suppose we have an input image *x* of size *H* × *W* with *C* channels, which is first transformed into a sequence of 1D tokens of length N=H × *W*, with each token having a dimension of *C*. This is performed using an embedding layer that maps each pixel in the image to a token vector:

(6) $$E\left(x\right)=\left[e_{1},e_{2},\ldots ,e_{N}\right]\in R^{\left(NxD\right)}$$

where *D* is the dimensionality of the token embedding, which is a hyperparameter of the model. Each token vector $e_{i}\in R^{D}$ is obtained by applying a linear projection followed by a non-linearity such as ReLU. Next, we apply a positional encoding for the linear projection of flattened layers to encode the spatial characteristics of the image. The positional encoding is defined in as follows:

(7) $$PosE\left(pos_{i},2j\right)=sin\left(pos_{i}/{10,000}^{\left(2j/D\right)}\right)$$

(8) $$PosE\left(pos_{i},2j+1\right)=cos\left(pos_{i}/{10,000}^{\left(2j/D\right)}\right)$$

where $pos_{i}$ is the position of the *i*-th token and j is the index of the dimension of the token embedding. The positional encoding is added to the token embeddings to obtain the input sequence for the transformer:

(9) $$X=E\left(x\right)+PosE$$

The next module is the transformer encoder. The transformer consists of a stack of L identical blocks, each containing a multi-head self-attention mechanism (MH) and a feedforward network also referred to as multi-layer perceptron (MLP). The attention mechanism allows the model to focus on relevant parts of the image while suppressing noise and irrelevant regions. The MH is defined by

(10) $$MH\left(V,Q,K\right)=join\left(hd_{1},hd_{2},\ldots ,hd_{h}\right)W^{O}$$

where $V,K,Q\in R^{\left(Nxd\right)}$ are the value, key, and query matrices, correspondingly, and d is the dimensionality of the attention space, which is typically smaller than *D*. $hd_{i}=Attention\left(VW_{i}^{V},QW_{i}^{Q},KW_{i}^{K}\right)\in R^{\left(Nxd/h\right)}$ is the *i*-th attention head, where $W_{i}^{Q},W_{i}^{K}$, and $W_{i}^{V}$ are learnable projection matrices, and $Attention\left(V,Q,K\right)=softmax\left(QK^{T}/sqrt\left(d\right)\right)V$ is the scaled dot-product attention function. The output of the multi-head attention mechanism is concatenated and projected using a learnable matrix $W^{O}$ to obtain the outcome of the block. The feedforward network is a two-layer MLP with a GELU activation function, defined as follows:

(11) $$FFN\left(Img\right)=GELU\left(ImgW_{1}+b_{1}\right)W_{2}+b_{2}$$

where $W_{1},W_{2}\in R^{\left(dx4d\right)}$ and $b_{1},b_{2}\in R^{4d}$ are learnable parameters. The output of the transformer is obtained by applying *L* blocks of the multi-head self-attention mechanism and feedforward network to the input sequence *X*:

(12) $$Y=Transformer\left(X\right)=Block_{L}\left(Block_{L-1}\left(\cdots \left(Block_{1}\left(X\right)\right)\right)\right)$$

### 3.5. Classification

Finally, once the dependencies between features and labels have been modeled using the Transformer encoder, a classifier is employed to make the final predictions for the labels. An independent feedforward network $\left({FFN}_{i}\right)$ is employed for final label assignment. $\ell_{i}^{,}.$${FFN}_{i}$ contains two linear layers.

(13) $$l\hat{a}b=FFN_{\ell_{i}}=softmax\left(Relu\left(\ell_{i}.wic+b_{j}\right)+b_{i}\right)$$

The loss function employed is categorical cross-entropy, and the optimization is performed using the Adam optimizer. In this study, a pre-trained vision transformer (ViT) model, initially trained on the ImageNet dataset, was employed as the foundation. Subsequently, the model underwent a fine-tuning process utilizing MRI images.

## 4. Evaluation Metrics

Various assessment metrics are employed to evaluate the proposed methodology. The list includes the confusion matrix, precision, recall, F-1 score, and micro-averaged accuracy. A confusion matrix is a tabular representation commonly employed for assessing the effectiveness of a classification model. The table displays the actual classification results against the predicted results of the model. A confusion matrix for a multiclass classification problem is similar to the binary confusion matrix, but it includes more than two classes. The confusion matrix for a multiclass problem is a square matrix, where each row corresponds to the instances predicted for a specific class, while each column represents the instances belonging to a particular actual class. The diagonal elements of the matrix indicate the number of correctly predicted samples, whereas the off-diagonal elements correspond to instances that were classified incorrectly.

For a multiclass classification problem, precision, recall, and F1-score can be defined based on the confusion matrix.

Precision quantifies the proportion of instances classified as positive that are genuinely positive. For class *i*, precision is defined as follows:

(14) $$precision_{i}=\frac{TP_{i}}{TP_{i}+FP_{i}}$$

Recall measures how many of the actual positive instances are predicted as positive. For class i, recall is defined as follows:

(15) $$recall_{i}=\frac{TP_{i}}{TP_{i}+FN_{i}}$$

$TP_{i}$ refers to the count of correctly identified instances belonging to class *i* as true positives, $FP_{i}$ represents the count of false positives for respective class *i*, and $FN_{i}$ represents the instances of false negatives for class *i*.

The F1-score is a metric that strikes a balance between precision and recall by calculating their harmonic mean. For class *i*, the F1-score is defined as follows:

(16) $$F1-score_{i}=2\times \frac{precision_{i}\times recall_{i}}{precision_{i}+recall_{i}}$$

## 5. Result and Analysis

**Figure 3:**
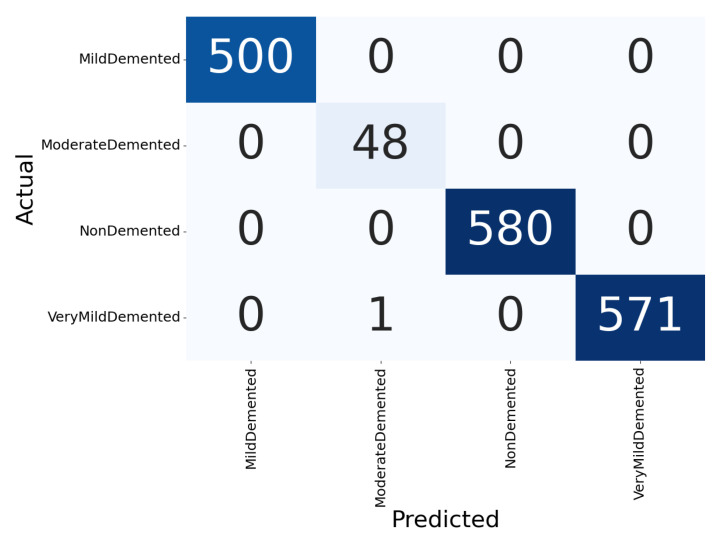
Confusion matrix for Alzheimer’s classification.

Figure 3 illustrates the confusion matrix of the proposed classification model for a four-class Alzheimer’s detection problem. The first row of the matrix represents the predicted instances for the Mild-Demented class. The model predicted this class correctly 500 times and incorrectly predicted instances of other classes as Mild-Demented zero times. The second row represents the predicted instances for the Moderate-Demented class. The model predicted this class correctly 48 times and incorrectly predicted instances of other classes as Moderate-Demented zero times. The third row represents the predicted instances for the Non-Demented class. The model predicted this class correctly 580 times. The fourth row represents the predicted instances for the Very Mild-Demented class. The model predicted this class correctly 571 times and incorrectly predicted instances of other classes as Very-Mild-Demented one time. This confusion matrix shows that the model has high accuracy for predicting all the classes. The model tends to correctly predict the Mild-, Moderate-, and Non-Demented classes.

**Figure 4:**
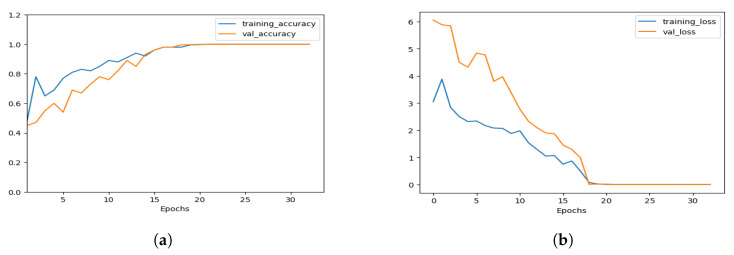
Curves depicting the loss and accuracy during the training and validation stages. (**a**) Accuracy curves. (**b**) Loss curves.

In Figure 4, the loss-accuracy curves for the training process are depicted, as well as the validation for Alzheimer’s detection. Loss curves are an important tool for monitoring the training and testing performance of machine learning models. The loss function measures how far off the predicted values are from the actual values. The goal is to find the model that minimizes this difference.

During the training process, the model is optimized to minimize the loss function by adjusting its parameters. The loss curves track the value of the loss function over the training and validation datasets as the model is trained over multiple epochs.

The validation loss curve shows how the value of the loss function changes during the training process on a separate dataset, called the validation dataset. The validation dataset is employed for the evaluation of the model’s capabilities on data that are not part of the training dataset. As the model learns from the training dataset, it also performs well on the validation dataset.

**Figure 5:**
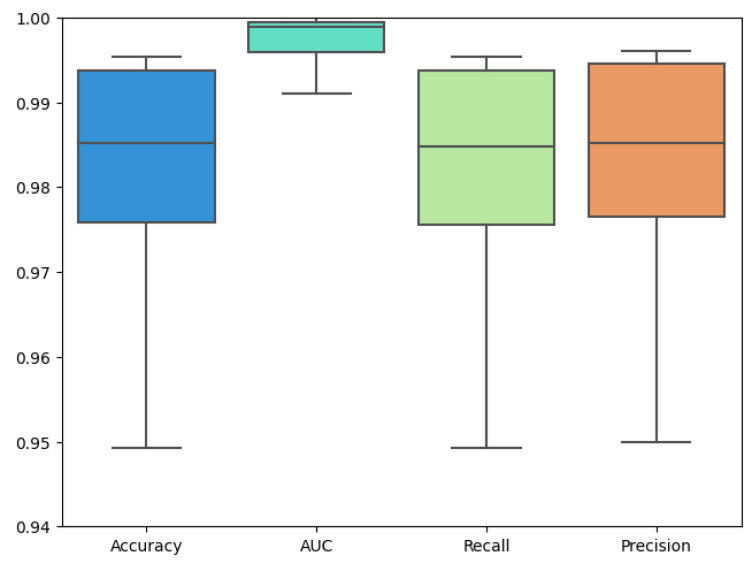
Boxplot for different evaluation metrics.

Figure 5 shows boxplots for different evaluation metrics of the Alzheimer’s detection process. Boxplots can provide a quick visual summary of the variability of values in a training process. They show the median, upper and lower quartiles, and minimum and maximum values.

Table 1 represents the results of Alzheimer’s detection on each stage. The model’s performance is evaluated using three common metrics for classification problems: precision, recall, and F1-score. Looking at the table, we can see that the model has excellent performance for all four classes, as indicated by high precision, recall, and F1-scores. These results suggest that the model has learned to accurately classify AD patients into their respective stages with high accuracy.

**Figure 6:**
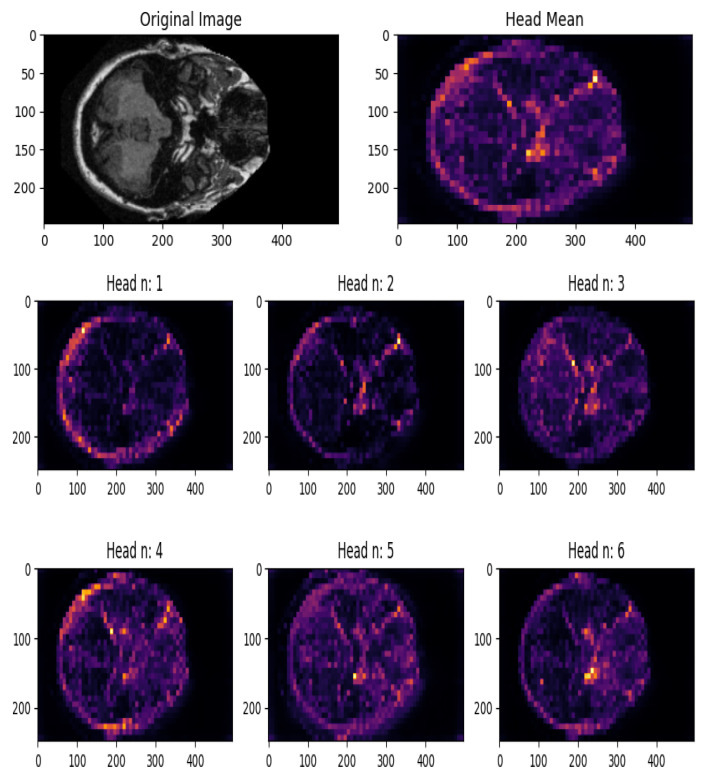
Attention head map visualization.

A transformer module comprises numerous heads, and each head projects the input data to unique sub-spaces in a transformer block. This enables every single head to focus on diverse portions of the image. Hence, it is logical to display each attention head map individually to understand what each head is focusing on. Figure 6 shows the attention head maps for a sample mild AD image. The top row of the headmap represents the mean of all the attention head maps, providing an overall view of the regions that the model attends to the most. The second and third rows show individual attention head maps, each corresponding to a specific attention head in the network. These individual maps reveal the model’s attention patterns for specific aspects or features in the image. The underlying motivation behind this visual interpretation is to gain insights into the model’s reasoning process and understand which regions or features are critical for detecting mild Alzheimer’s disease. By visualizing the attention head maps, researchers can identify regions in the image that receive higher attention weights, indicating their importance in making the final prediction.

**Figure 7:**
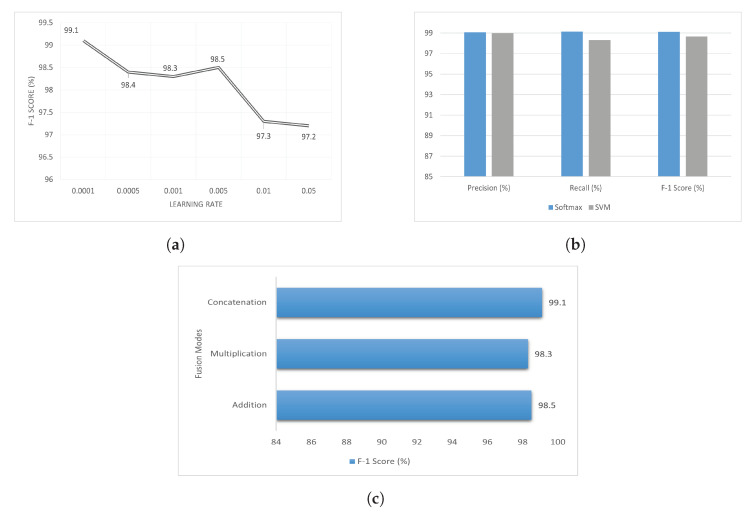
Ablation study results for different parameters. (**a**) Learning rate. (**b**) Classifiers. (**c**) Feature fusion.

An ablation study is a technique employed to evaluate the significance of various features or components within a model. By selectively altering or eliminating specific elements of the model, researchers can examine how these modifications affect the model’s performance. Through this process, valuable insights can be obtained regarding the relative importance or influence of each individual factor. Different factors are analyzed for the optimized results of the presented technique. Figure 7 shows the results of different choices. Various learning rates are examined during the training phase of the proposed method. The learning rate is denoted as lr, with lr taking values from the set {0.001,0.01,0.005,0.05,0.0001,0.0005}. An lr of 0.0001 gives the best results. Two classifiers, namely softmax and support vector machine (SVM), are also compared. Softmax gives slightly better results. Feature fusion can be performed in different ways in the transformer architecture. We employed three different ways of feature fusion, namely addition, multiplication, and concatenation. Feature fusion using concatenation outperformed the other two methods.

The comparison table (Table 2) represents the performance evaluation of different algorithms for AD detection, using their precision, recall, and F1-score as criteria. All the reported results are on same dataset. The table shows the outcomes of five different methods, including four previously published methods and the proposed method. The proposed method outruns the rest of the techniques in terms of accuracy, precision, recall, and F1-score, attaining a precision of 99.99%, recall of 99.99%, and F1-score of 99.99%. This suggests that the proposed method has learned to classify AD with high accuracy and precision and can identify a high proportion of true positive instances while minimizing the number of false negatives.

The proposed deep learning model demonstrates remarkable performance in detecting Alzheimer’s disease using MRI images. The confusion matrix analysis reveals high accuracy in predicting the Demented and Non-Demented classes. The loss-accuracy curves exhibit effective training and validation performance, showcasing the model’s ability to generalize well. Moreover, the attention head maps provide insights into the regions of focus for the vision transformer during classification. The ablation study highlights the importance of various choices for optimizing the proposed technique. Comparison with other methods demonstrates the superiority of the proposed approach in Alzheimer’s detection on the chosen dataset. However, the model’s performance may vary depending on the dataset and modality employed, warranting further research for broader applicability.

## 6. Limitations and Future Recommendations

This research demonstrates promising results in an academic setting, but the absence of clinical validation and real-world testing hinders the practical applicability of the proposed method in clinical diagnosis and treatment. Moreover, the research relies on a single publicly available Kaggle dataset, which might introduce dataset-specific biases and limit the generalizability of the proposed model to diverse populations or different acquisition protocols.

To validate the proposed method’s generalizability, future research should evaluate the model on multiple independent datasets with varied demographics and imaging protocols, spanning different populations and geographical regions. Conducting clinical trials and collaborating with medical experts for expert validation would strengthen the proposed method’s credibility and potential adoption in real clinical settings. Other AI and machine learning techniques can also be used for the focused problem [46,47]. Incorporating explainable AI techniques can enhance the model’s interpretability, allowing clinicians to understand the features contributing to the diagnosis and fostering trust in the AI system.

## 7. Conclusions

The proposed approach offers an AD detection method that utilizes a vision transformer architecture with MRI images. The methodology includes preprocessing the MRI images and inputting them into the vision transformer network for classification. By leveraging self-attention mechanisms, the network is trained on a Kaggle dataset of MRI images to learn discriminative features for AD detection.

The experimental findings illustrate the remarkable effectiveness of the proposed approach in AD detection using MRI, surpassing other published methods on the Kaggle dataset. However, it is crucial to acknowledge that performance may vary depending on the specific dataset and imaging modality utilized. As a result, future research endeavors should concentrate on devising effective techniques for AD detection encompassing diverse datasets and modalities. Furthermore, several alternative methods have undergone evaluation on different datasets and modalities, in addition to the proposed approach. These methods include DEMNET, EfficientNetV2B1, InceptionResnetV2, InceptionV3, and Acharya. These methods achieved varying levels of accuracy, precision, recall, and F1-score, depending on the dataset and modality used. An area that holds promise for future investigation is the exploration of multimodal approaches that integrate distinct modalities, such as MRI and fMRI, to enhance AD detection capabilities. Another area for future research is the development of methods that can handle imbalanced datasets, which is a common issue in AD detection due to the large number of healthy controls compared to patients. Moreover, research can be conducted to identify the most informative regions of the brain for AD detection, which can help in developing more accurate and efficient methods. Additionally, methods can be developed that can track disease progression and predict the onset of AD.

## Figures and Tables

**Table 1:** Evaluation metrics for each specific class.

Disease	Precision%	Recall%	F1-Score%
ND	100	100	100
VMD	99.99	100	99.99
MID	100	100	100
MOD	100	100	100

**Table 2:** Performance comparison on Kaggle dataset.

Methodology	Precision%	Recall%	F-1%
Kabir et al. (2021) [45]	92.78	90.78	0.94
EfficientNetV2B1	90.37	89.76	90.06
InceptionResnetV2	97.4	94.76	95.80
InceptionV3	98.13	97.72	98.05
Proposed	99.99	99.99	99.99

## Data Availability

Not applicable.
